# Experimental Investigation of Tool Wear and Machining Quality of BTA Deep-Hole Drilling in Low-Carbon Alloy Steel SA-5083

**DOI:** 10.3390/ma16206686

**Published:** 2023-10-14

**Authors:** Xubo Li, Chuanmiao Zhai, Wenqi He, Ye Lu, Bodong Zhang

**Affiliations:** 1 College of Mechanical Engineering, Baoji University of Arts and Sciences, Baoji 721016, China; 2 Shaanxi Key Laboratory of Advanced Manufacturing and Evaluation of Robot Key Components, Baoji 721016, China

**Keywords:** deep-hole machining, surface quality, machining precision, BTA drill, tool wear

## Abstract

Nuclear power tube plates are made from the high-strength, low-carbon alloy steel SA-5083, which has high values of toughness and plasticity, though it is forged with poor consistency and entails serious work hardening. It requires a large number of deep holes with a high machining accuracy and high surface quality to be processed. However, the quality of the processed holes is often not up to the standard of the Boring and Trepanning Association (BTA) for the deep-hole drilling of tube plates; this has led to deep-hole processing becoming a bottleneck in the manufacture of steam generators for the main equipment of nuclear power islands. The variation laws of the diameter, roundness, perpendicularity, roughness, microhardness, and residual stress in relation to the feed, speed, and drilling depth are explored in the macro- and micro-dimensions; also explored is the wear morphology of BTA drills. The internal influence mechanisms between them are revealed in order to provide a scientific basis for the control of surface quality and machining accuracy as well as the optimization of process parameters. Our research results indicate that the guide block wear is mainly concentrated at the top 1–2 mm and that the drilling depth and feed have a great influence on the machining diameter. The hole wall roughness is between 0.3 and 0.6 μm, the maximum microhardness is about 2.15 times the hardness of the matrix material, and the residual stress is compressive stress. With increases in the feed and drilling depth, the hole diameter and the roughness increase. With an increase in the speed, the roughness decreases and the compressive stress of the BTA deep-hole drilling wall increases.

## 1. Introduction

The steam generator of the main equipment of a nuclear power island contains a nuclear power tube plate, which is a heat exchange baffle with a radioactive heat carrier on the primary side and water on the secondary side. Its material is the high-strength, low-carbon alloy steel SA-5083, which has high values of toughness and plasticity, though it is forged with poor consistency and entails serious work hardening [1]. The deep-hole group drilled in the tube plate is used for the heat exchange, after the completion of the Inconel 690 tube bundle expansion tube. The deep-hole machining quality of the nuclear power tube plate directly affects the heat exchange efficiency and radiation resistance capacity of the nuclear power steam generator [2]. BTA deep-hole drilling is a high-efficiency deep-hole processing method which is used for processing holes with a high length-to-diameter ratio; thus, it is the main method for the deep-hole machining of nuclear power tube plates [3,4,5,6].

Due to the narrow chip evacuation space and long chip evacuation distance in deep-hole machining, the tool is always in a semi-closed room, and the rigidity of the processing system is poor; so, it is often difficult to break chips and evacuate them [7], and the tool wears down quickly [8,9,10], creating poor drilling stability [11,12] and other problems. Deep-hole machining is one of the most difficult processes in the manufacturing industry. Generally, cylindrical holes with a depth-to-diameter ratio L/D (L is the depth of the hole, and D is the hole diameter) greater than five are called deep holes. However, the depth-to-diameter ratio of deep holes in nuclear power tube plates exceeds 45, and it also requires high values for the surface quality and the machining accuracy. As a result, the quality of the deep-hole drilling of the nuclear tube plate often fails to meet the standard, which causes the hole processing of the tube plate to become a bottleneck in the manufacture of the main equipment used in nuclear power [13,14].

Machining quality includes machining accuracy and surface quality. For deep-hole machining, the machining accuracy mainly includes the hole diameter, straightness, and roundness, and the surface quality mainly includes the hole wall roughness, subsurface microstructure and hardness, and residual stress [15,16]. In the deep-hole processing of nuclear power tube plates, the diameter deviation of the hole affects the tightness of the expansion tube; the roundness affects the fit between the hole wall and the heat exchange pipes; the perpendicularity affects the uniformity of the distribution of the pipes; the roughness of the hole wall affects the actual contact area and the fit quality between the hole wall and the pipes; the metallographic structure and microhardness of the subsurface of the hole wall affect the corrosion resistance; and the residual stress of the hole wall affects the fatigue strength [2,17]. However, BTA deep-hole drilling relies on the tool’s own guide blocks to balance the cutting insert’s force to achieve self-guidance in deep-hole machining. The influence mechanism of deep-hole machining accuracy and surface quality is very complex [18,19].

The resultant radial force and tool system vibration are reduced by changing the angle of the cutting edge of the inserts [20,21]. Zhang et al. [22] investigated the influence of the offset of the cutting edge of a gun drill on the straightness of the deep hole, aiming at the problem of easy deviation in the deep-hole drilling of Inconel 718 material. Tnay et al. [23] studied the effect of the insert’s cutting edge angle on the chip removal efficiency as well as the cutting heat dissipation. The machining hole accuracy and surface quality are affected not only by the cutting inserts but also by the burnishing of the guide blocks [24]. Matsuzaki et al. [25] demonstrated through experimental research that increasing the number of guide blocks can increase accuracy in machining holes. Felinks et al. [26] determined that the vibration of the deep-hole machining tool system was affected by the chamfer of the guide block. Lopez de Lacalle et al. [27] used a spindle motor torque signal to monitor drilling status to improve drilling performance. Si et al. [28] established a model of the relationship between the spindle vibration signal and the roundness of the machined deep hole in order to predict the roundness. Kong et al. [29] designed a magneto-rheological fluid device to adjust the stiffness and damping of the drilling tool system to control the drilled hole roundness. Yu [30] developed a wedge-shaped self-centering device acting between the drilling tube and the hole wall to improve the straightness of the machining hole. Furthermore, the quality of deep-hole machining is closely related to the drilling process parameters in addition to the drilling depth [31]. Neo et al. [32] explored the variation laws of the gun drill guide block’s wear, drilling axial force and torque, and drilled hole roundness in relation to the drilling depth.

BTA deep-hole machining is different from ordinary metal cutting. It is a composite machining method of insert cutting and guide block extrusion which belongs to flexible machining. It must rely on the perfect fit of the insert and guide block to ensure the self -centering of the drill. The technical threshold is high, and the precise control of the machining quality is difficult. In addition, during the drilling process, the BTA drill is in a high-pressure, saturated cutting fluid, and the wear mechanism of the tool is also different from that of the other machining methods.

Although previous researchers have conducted a lot of work on the drilling accuracy and surface quality of deep holes, the database of the BTA deep-hole drilling process system is not complete. Research on the BTA deep-hole drilling process for nuclear power tube sheets with extremely strict requirements regarding machining accuracy and surface quality is even more scarce. This paper studies the variation laws of deep-hole machining accuracy and hole wall surface quality in relation to the drilling feed, drilling speed, and drilling depth in the macro- and micro-dimensions, as well as the wear form of the BTA drill, and it reveals the internal influence mechanism between them. It provides a scientific basis for the dimensional accuracy and surface quality control of deep-hole machining, the design optimization of the BTA drill, and the optimization of the drilling process parameters. Additionally, it lays a theoretical foundation for the development of precision deep-hole machining technology.

Innovation points: (a) In the designed experiments, the variation laws of the diameter, roundness, perpendicularity, roughness, microhardness, and residual stress in relation to the feed, speed, and drilling depth are systematically explored in the macro- and micro-dimensions of BTA deep-hole machining. (b) By obtaining the wear morphology of BTA deep-hole cutting tools through SEM and an energy spectrum analysis while drilling with the low-carbon alloy steel SA-5083 with Inconel 690 overlay welding for nuclear power tube sheets, the wear mechanism and type of the BTA drill are revealed.

Organization: This paper consists of four sections, namely Introduction, Experiments and Measurements, Results and Discussions, and Conclusions. (a) The Section 1 introduces the research background and its significance, analyzes the current status of research, clarifies existing problems and gaps, and provides the main contributions, research objectives, innovation points, and paper organization. (b) The Section 2 describes the machining method and workpiece material, the experimental equipment and tools, and the experimental procedures and measurements. (c) In the Section 3, the experimental results are presented, discussed, and analyzed. (d) The Section 4 presents the research results and conclusions of the paper.

## 2. Experiments and Measurements

### 2.1. Machining Method and Workpiece Material

The deep-hole processing of the third-generation AP1000 nuclear power steam generator tube plate is as follows: 20,050 deep holes with a diameter of 17.73 mm need to be uniformly processed on a tube plate with a diameter of 4500 mm and a thickness of 800 mm. The material of the tube plate is high-strength, low-carbon alloy steel SA-5083 (Tongxiang Metal Materials (Shanghai) Co., Ltd, Shanghai, China); one side is also surfaced with Inconel 690 (Tongxiang Metal Materials (Shanghai) Co., Ltd, Shanghai, China) at a thickness of about 8 mm. The chemical element composition of the substrate and surfacing layer materials is shown in Table 1 [2]. The shortest cycle for the deep-hole processing of a nuclear power tube sheet is three months. If a hole fails to meet the design requirements, the whole drilled tube sheet will be scrapped, and the overall construction progress of the nuclear power plate will be seriously affected. Therefore, the deep-hole drilling of a nuclear power tube plate has strict requirements with regard to machining efficiency, accuracy, and the surface quality of the holes.

**Figure 1:**
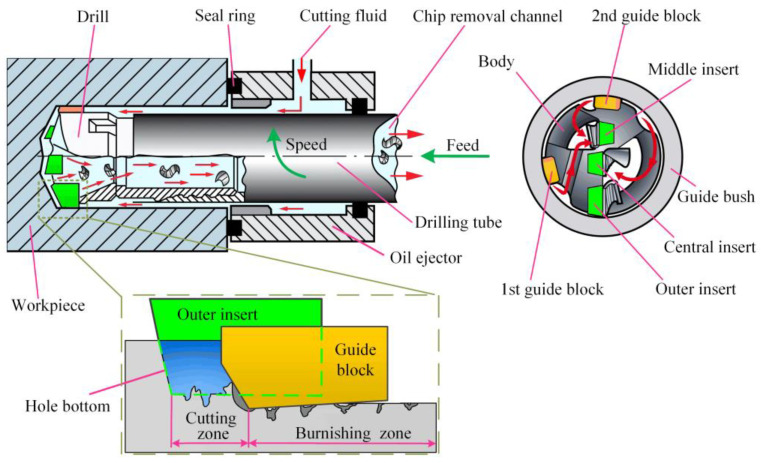
Machining principle of BTA deep-hole drilling system.

The principle of the BTA deep-hole processing system is shown in Figure 1. The cutting inserts of the BTA drill are arranged in a dislocated manner and include the central insert, the middle insert, and the outer insert. The guide blocks are distributed outside of the drill. The BTA deep-hole drilling is completed with the cutting of the workpiece material by the inserts and the secondary burnishing by the guide blocks. The guide blocks are responsible for balancing the tangential and radial resultant force from the cutting inserts, extruding the hole wall from the cutting inserts, completing the self-centering of the machining, and improving the accuracy and surface quality of the hole wall.

### 2.2. Experimental Equipment and Tools

**Figure 2:**
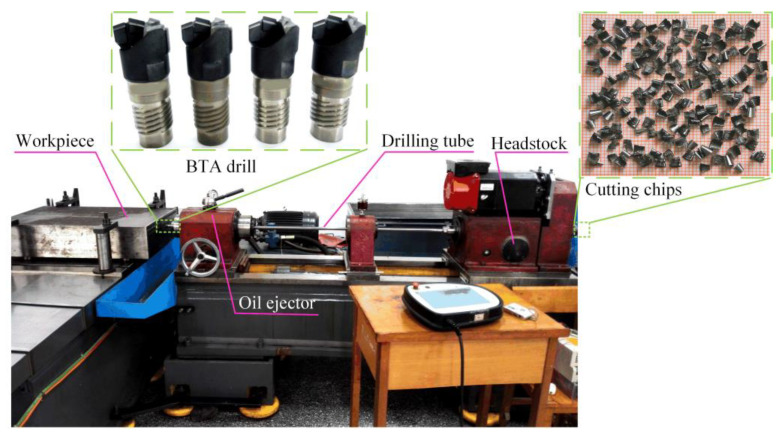
Experimental platform of BTA deep-hole drilling.

The experimental platform used for deep-hole processing is shown in Figure 2. The drilling tube length is 1500 mm, with an allowable drilling depth of 1000 mm. The spindle motor is an AC servo motor with a speed of 2500 r/min and a power of 7.5 kW. The cutting oil is a special cutting fluid for deep-hole drilling containing extreme pressure additives; the supply pressure is 6 MPa, and the flow is 90 L/min.

The workpiece material for the deep-hole drilling test is the same as that of the tube plate of the third-generation AP1000 nuclear power plant. Its dimensions are 800 mm in length, 170 mm in height, and 420 mm in width. The tool used in the experiment is a BTA drill of ∅17.75 mm; its geometric parameters are illustrated in Table 2. The manufacturer of the BTA drill is Chengdu Tool Research Institute Co., Ltd., in Chengdu, China. The hard alloy grade of the cutting insert is P20, and the hard alloy grade of the guide block is GWC. The recommended cutting speed for drilling low-carbon alloy steel is 40–120 m/min, and the drilling feed is 0.02–0.12 mm/r.

### 2.3. Experimental Design and Measurements

The control variable method was used to deeply investigate the influence of the drilling feed, drilling speed, and drilling depth on the quality of the machined hole. When the drilling speed was 1200 r/min, the drilling feeds were taken to be 0.04, 0.06, 0.08, 0.1, and 0.12 mm/r, respectively. When the drilling feed was 0.08 mm/r, the drilling speeds were taken to be 800, 1000, 1200, 1400, and 1600 r/min, respectively. Each group of experiments was repeated three times.

**Figure 3:**
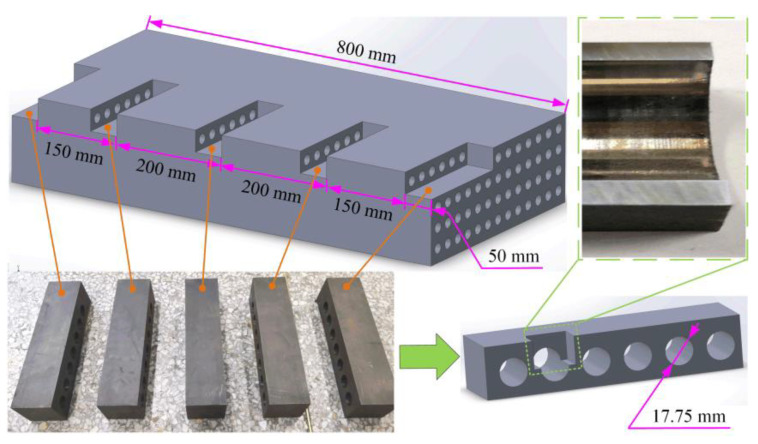
Cutting specimen for hole size accuracy measurement.

After drilling, the wear status of the BTA drill was observed using the KEYENCE VHX-5000 electron microscope (KEYENCE CO., LTD., Osaka, Japan ), and the morphology of the processed holes was observed using the Olympus IPLEX G Lite endoscope (Olympus (Shenzhen) Industrial Co., Ltd, Shenzhen, China). Furthermore, to study the effect of drilling depth on the quality of the machined holes, the drilled workpiece was cut along the drilling depth direction at hole depths of 50, 200, 400, 600, and 800 mm, respectively; the thickness of the specimen was 50 mm, as shown in Figure 3. The hole diameter, roundness, and perpendicularity of the cut specimens were measured on the Zeiss CONTURA G27 CMM (Carl Zeiss AG, Oberkochen, Germany), and the diamond probe of the CMM was measured within the range of 5 to 45 mm from the specimen. The variation law of deep-hole machining accuracy with the drilling parameters of nuclear power tube plate specimens was attained.

Next, the specimens were cut horizontally along the hole diameter and sampled for measurement. The morphology of the hole wall was observed under the OLYMPUS-BX51M microscope (Olympus (Shenzhen) Industrial Co., Ltd., Shenzhen, China), and the hole wall roughness was measured along the drilling feed direction using a Surtronic S-100 roughness measuring instrument (Taylor Hobson Ltd., Leicester, UK). The residual stress of the hole wall was tested using an Xstress 3000 (G2) X-ray stress analyzer (Stresstech Oy, Jyväskylä, Finland). 

Finally, the surface cut along the diameter of the hole was polished, and 4% nitric acid alcohol solution was used to corrode it. The corrosion time was 3 min. After the corrosion, it was cleaned with anhydrous ethanol, and the metallographic structure of the hole wall subsurface layer was observed. The Wilson Tukon 2500 automatic Vickers hardness tester (WILSON Co., Ltd., Minneapolis, MN, USA) was used to detect the microhardness distribution law in the subsurface layer of the hole wall. To obtain accurate measurement results and prevent damage to the edge area of the hole wall, indentations were made at the subsurface layer 10 μm away from the hole wall, followed by an interval of 20 μm; the total measurement depth was 300 μm. The experimental test results were fitted using quadratic polynomials to obtain their changing trends and to provide the fitting index R-square. The hole wall surface quality and accuracy of the deep-hole machining of the nuclear power tube plate specimen with the drilling parameters were obtained.

## 3. Results and Discussions

### 3.1. Tool Wear

**Figure 4:**
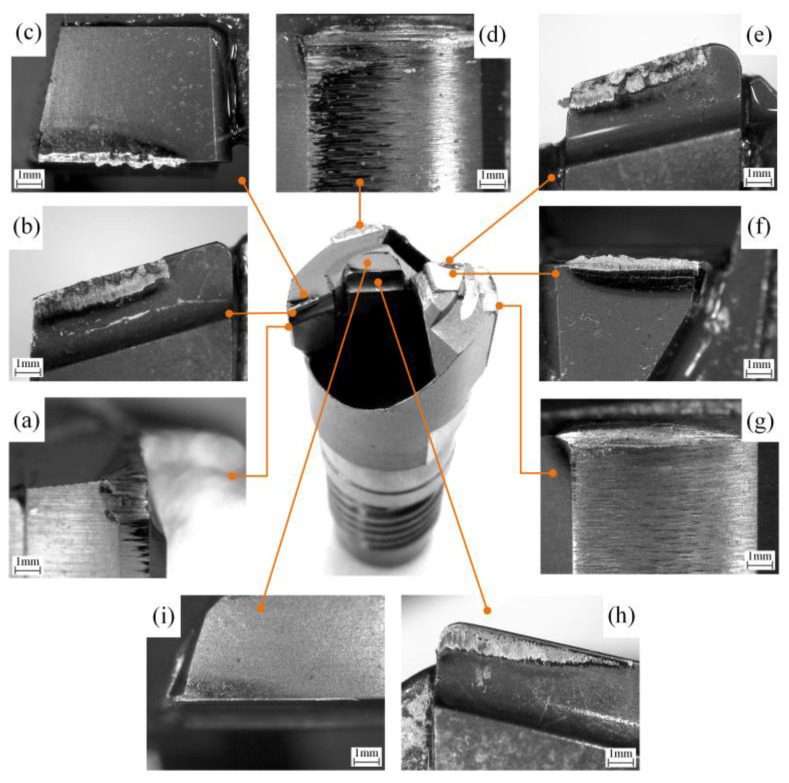
Wear morphology of BTA drill for deep-hole machining: (**a**) edge sizing blade of outer insert; (**b**) the rake face of outer insert; (**c**) the flank of outer insert; (**d**) the first guide block; (**e**) the rake face of middle insert; (**f**) the flank of middle insert; (**g**) the second guide block; (**h**) the rake face of central insert; and (**i**) the flank of central insert.

The wear pattern of the BTA drill is different from that of ordinary cutting tools. In addition to the wear of the three cutting inserts, there are also two guide blocks that create squeezing friction with the hole wall. The wear morphology of the drill under an electron microscope was magnified 50 times, as shown in Figure 4.

The rake face wear of the cutting insert of the BTA drill is a crescent shape. Due to the friction between the chips and the rake face when it flows out, high temperatures will appear in this area, resulting in a decrease in red hardness. At the same time, it will withstand the compression of the chip and form an arc groove under continuous scratching. During the cutting process, the flank of the cutting insert undergoes compression and friction contact with the bottom of the drilled hole, resulting in high contact stress and a high temperature. This causes the flank of the tool to become a worn edge with a zero-degree flank angle. Due to the different cutting speeds, the material removal productivity of the three cutting inserts is greater for the outer insert than for the middle insert, which is greater than that for the center insert. The temperature in the cutting area of each cutting insert is greater for the outer insert than for the middle insert, which is greater than that for the center insert, resulting in a greater wear of the outer insert than of the middle insert, which is greater than that of the center insert. In addition, all three cutting edges of the cutting inserts are bonded with a built-up edge, which is the most severe on the middle insert and has the greatest thickness.

The wear of the outer insert edge sizing blade is concentrated at a distance of 1–1.5 mm from the main cutting edge of the outer insert, which is the lagging area of the guide block relative to the outer cutting insert. The wear of the edge sizing blade along the radial direction of the drill forms deep grooves. When the hole wall undergoes the guide block extrusion contact, due to the fluidity of the metal material, the hole wall material in the axial area to be extruded accumulates and bulges at the front of the guide block, resulting in an increase in the cutting area of the edge sizing blade. At the same time, during the material flow process in the cutting zone, processing strengthening occurs and its strength increases. In addition, the outer insert sizing blade is located in the edge area, with low strength, resulting in the increasing wear of the sizing blade.

The guide block wear is mainly concentrated at the top 1–2 mm, and the wear of the other contact surfaces is relatively small. The wear of the first guide block is more severe than that of the second guide block. Due to the existence of inverted cones in the design of the guide block and the drill bending, the contact inclination angle between the guide block and the hole wall increases, reducing the contact area and high stress area. So, the guide block wear is concentrated at the top. When the drill rotates at a high speed, the other areas of the guide block are in a high-pressure cutting fluid, and dynamic pressure oil films are formed in the non-high-stress contact area, reducing the intensity of the extrusion friction.

**Figure 5:**
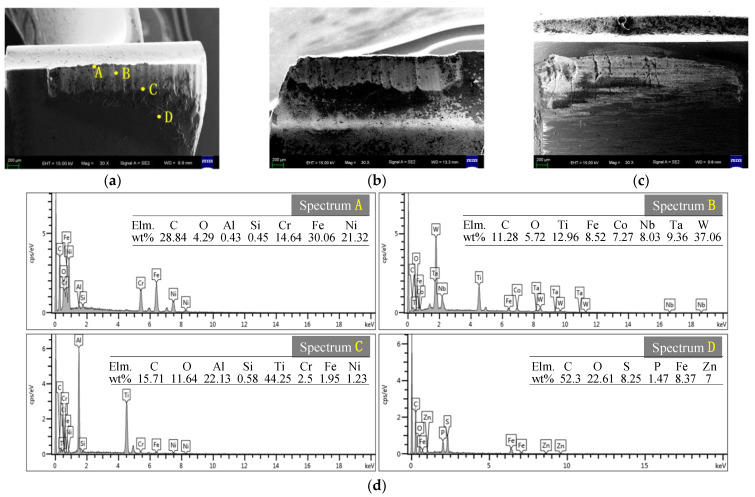
SEM morphology of BTA drill wear area and energy spectrum: (**a**) the flank of outer insert; (**b**) the rake face of outer insert; (**c**) first guide block; and (**d**) energy spectrum of the flank of outer insert.

The wear morphology of different areas of a severely worn drill under a scanning electron microscope is shown in Figure 5. When the insert cuts, the temperature increases and the hardness decreases. The hard points in the workpiece material continuously slide against the insert’s cutting edge, forming a striped wear band on the flank of the outer insert, as shown in Figure 5a. The crescent depression on the rake face of the insert can lead to a decrease in the cutting edge strength, which is prone to edge collapse. The strength of the outer insert’s cutting edge area is low, the cutting speed is high, and there is serious boundary wear, as shown in Figure 5b. Due to the high contact stress between the first guide block and the hole wall, fatigue wear and crack formation may occur on the contact surface of the guide block after machining multiple holes, as shown in Figure 5c. An element energy spectrum EDS analysis was conducted on different areas of the flank of the outer insert, and the chemical element distribution and mass percentage of the materials at different positions are shown in Figure 5d.

Point A belongs to the bonding zone of the built-up edge, and the main constituent elements of point A in the energy spectrum are Fe, C, Ni, and Cr, in that order. According to Table 1, the elements Ni and Cr are the main components of Inconel 690 in the weld overlay layer of the nuclear power tube sheet. Although the thickness of the nickel base layer is only 8 mm, accounting for only 1% of the single hole depth of the nuclear power tube sheet, the nickel base material still adheres to the cutting edge, indicating a strong adhesion between the nickel base material and the cutting insert [9].

Point B belongs to the wear zone of the outer insert, and the main elements in energy spectrum B include Fe and Ti in addition to the matrix material elements of the insert. Fe is the main component of the workpiece matrix material, and Ti is the main component of the TiAlN coating on the drill. Under high-temperature and -pressure conditions, the elements of the cutting insert matrix, coating, and workpiece material diffuse into each other, changing the original chemical composition and properties of the material and exacerbating the diffusion wear.

Point C belongs to the edge of the wear band on the outer insert flank, and the main components of the energy spectrum C are Ti, Al, C, O, and Fe, in that order. Under the high-speed friction between the hole bottom and the flank, high-temperature conditions are formed, promoting oxidation reactions in the edge area of the wear band. The generated Al_2_O_3_ and FeO_x_ have high levels of hardness and thermal stability, which slow down the tool wear.

Point D is located in the rear area of the wear zone, and the main elements in the energy spectrum D are C, O, Fe, S, P, in that order. S and P are the main components of the organic sulfides and organic phosphides in the extreme pressure additives of deep-hole drilling cutting fluid. Therefore, the black layer formed in the rear area of the wear zone is the product of the reaction and the accumulation of the cutting fluid.

### 3.2. Macromorphology

**Figure 6:**
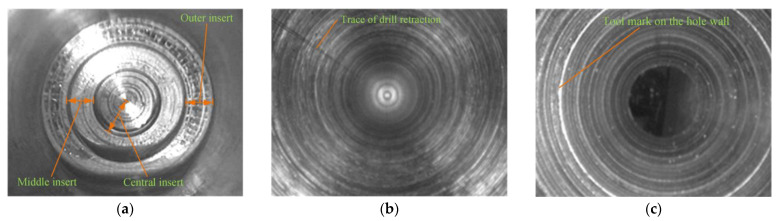
Morphology of machined holes: (**a**) hole bottom; (**b**) normal hole wall; and (**c**) hole wall of tool system chatter.

The morphology of the drilled hole wall and bottom observed by the endoscope is exhibited in Figure 6. Figure 6a shows the hole bottom formed by the cutting insert, and the cutting area of the outer insert, middle insert, and central insert as well as the cutting width of each cutting insert along the radial direction can be clearly distinguished. Hole machining involves tool rotation and the axial feed; spiral tool marks are generated on the hole wall. Due to the squeezing effect of the guide blocks, the waviness of the hole wall is relatively small. However, there is a tool retraction mark along the axial direction of the hole wall, as shown in Figure 6b, because the outer insert of the BTA drill has a narrow sizing blade, resulting in scratches being left on the hole wall during the BTA drill retraction. Figure 6c shows the hole wall formed after the tool chatter. The drilling tube rigidity is poor, and the cutting tool system is prone to vibration, resulting in an increase in the radial runout of the BTA drill and an increase in the waviness and roughness of the hole wall.

### 3.3. Diameter

**Figure 7:**
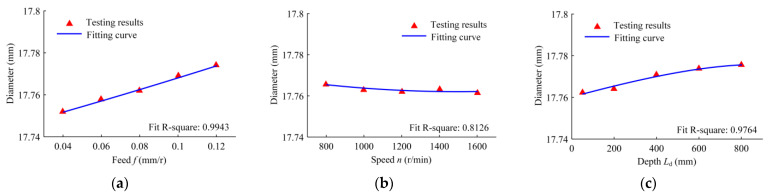
Variation in the drilling hole diameter with the following drilling parameters: (**a**) the drilling feed range from 0.04 to 0.12 mm/r, when the speed is 1200 r/min and the depth is 50 mm; (**b**) the drilling speed range from 800 to 1600 r/min, when the feed is 0.08 mm/r and the depth is 50 mm; and (**c**) the drilled depth range from 50 to 800 mm, when the speed is 1200 r/min and the feed is 0.08 mm/r.

The variation laws of the drilling hole diameter in relation to the drilling feed, rotational speed, and drilling depth are illustrated in Figure 7. When the drilling feed increases, the cutting force increases, the insert’s cutting force increases, the positive pressure of the guide block increases, the plastic deformation from the guide block’s burnishing increases, and the hole diameter increases. The feed increased from 0.04 mm/r to 0.12 mm/r, and the hole diameter increased by 22 μm. When the speed increases, the time for the extrusion contact deformation of the hole wall at the same particle point decreases, which brings about a reduction in the extrusion deformation and hole diameter. For this reason, the speed has little effect on the cutting force, and the guide blocks have a weak influence on the extrusion deformation. With the increase in the machining hole depth, the overhang length of the drilling tube increases, the drilling tube bending increases, the contact angle between the guide blocks increases, the contact area decreases, and the burnishing deformation increases; thus, the drilling hole diameter increases. The hole diameter increased by 13 μm when the drilling depth increased from 50 mm to 800 mm.

### 3.4. Roundness

**Figure 8:**
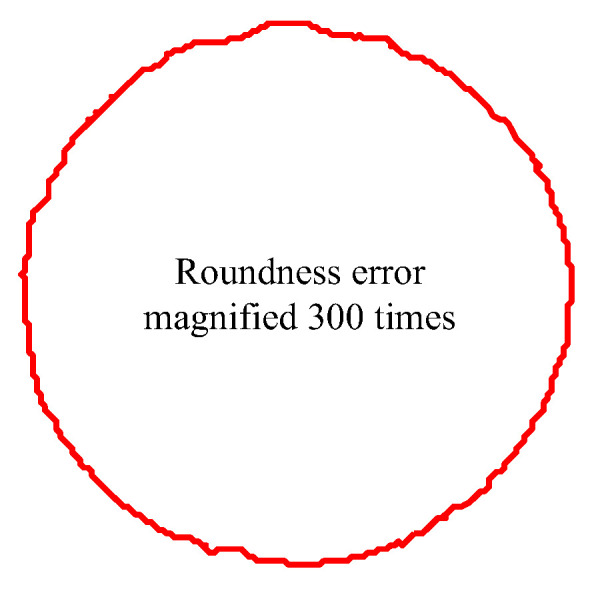
Roundness error profile of machining deep hole.

Roundness is an important criterion for evaluating the accuracy of a machined hole. It is the distance between the circumscribed circle and the inscribed circle of the actual profile of the machined hole, and it indicates how close the cross-section of the workpiece is to the theoretical circle. Under the machining process parameters of a speed of 1200 r/min and a feed of 0.08 mm/r, the roundness error of the 50 mm drilling depth was 6.2 μm. When the drilling hole diameter was unchanged, the profile of the roundness error was magnified by 300 times, as revealed in Figure 8.

**Figure 9:**
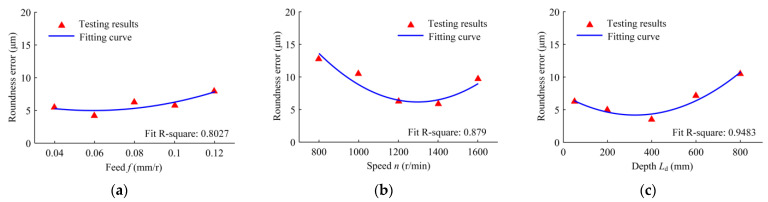
Variation in the hole roundness error with the following drilling parameters: (**a**) the drilling feed range from 0.04 to 0.12 mm/r, when the speed is 1200 r/min and the depth is 50 mm; (**b**) the drilling speed range from 800 to 1600 r/min, when the feed is 0.08 mm/r and the depth is 50 mm; and (**c**) the drilled depth range from 50 to 800 mm, when the speed is 1200 r/min and the feed is 0.08 mm/r.

Figure 9 shows the roundness errors of the machined holes for different drilling feeds, speeds, and depths. The effect of the drilling feed on the roundness error is small. With the increase in the feed, the roundness error increases. Compared with the feed, the speed has a greater effect on the roundness error. With the increase in speed, the roundness error first decreases and then increases. With the increase in drilling depth, the roundness error decreases first and then increases. When the feed increases, the drilling force and the force fluctuation increase and the roundness error increases. As a result of the tool system in BTA drilling being a dynamic change process, the roundness is affected by the vibration characteristics of the drilling tool. The drilling speed directly affects the excitation frequency of the deep-hole machining system, and drilling depth affects the natural frequency of the drill, which has an effect on the hole roundness.

### 3.5. Perpendicularity

The hole perpendicularity is affected not only by the manufacturing accuracy of the drilling equipment, but also by the burnishing contact state between the guide blocks and the hole wall. The support sealing sleeve of the oil ejector, the center bracket, and the end of the spindle box connecting the drilling tube must have a high degree of coaxiality, and the axis established by the three must be perpendicular to the end face of the workpiece, otherwise it will lead to hole deflection [33]. Moreover, it is also necessary to control the matching tolerance of the inner diameter of the guide bush and the BTA drill. If the matching tolerance is large, there will be a positional deviation during the entrance of the drill. With the increase in the drilling depth, the accumulated errors will cause the perpendicularity of the drilled hole to be out of the tolerance limit.

**Figure 10:**
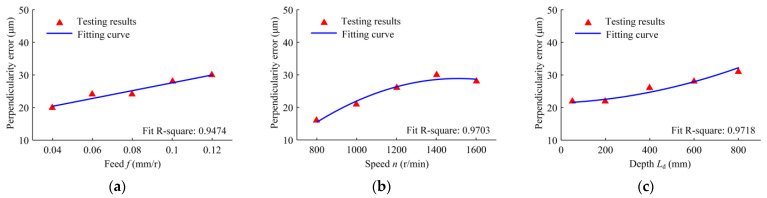
Variation in the hole perpendicularity error with the following drilling parameters: (**a**) the drilling feed range from 0.04 to 0.12 mm/r, when the speed is 1200 r/min and the depth is 50 mm; (**b**) the drilling speed range from 800 to 1600 r/min, when the feed is 0.08 mm/r and the depth is 50 mm; and (**c**) the drilled depth range from 50 to 800 mm, when the speed is 1200 r/min and the feed is 0.08 mm/r.

Figure 10 indicates the perpendicularity error of the machined holes in the range of the testing depth of 5–45 mm under different conditions of feed, speed, and depth. The perpendicularity error has a linear relationship with the feed. With an increase in the feed, the perpendicularity error increases. As the speed increases, the perpendicularity error increases; its growth rate is larger when the speed is low, and the growth rate decreases with the further increase in speed. With the increase in drilling depth, the perpendicularity error and its growth rate increase. When the drilling feed increases, the cutting force increases and the extrusion deformation on the hole wall increases, resulting in the increase in the perpendicularity error. As the drilling depth increases, the cumulative error of the perpendicularity increases.

### 3.6. Roughness

**Figure 11:**
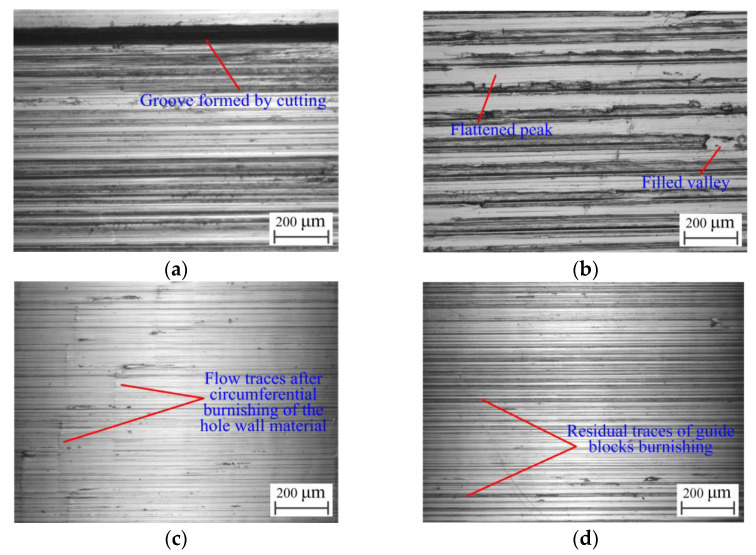
Hole wall under different conditions: (**a**) formed by the cutting insert; (**b**) formed by inadequate extrusion of the guide blocks; (**c**) formed by normal extrusion of the guide blocks; and (**d**) formed by excessive extrusion of the guide blocks.

The drilling hole wall is not the trace left by the cutting inserts; it is the comprehensive surface of the cutting by the edge sizing blade of the outer insert and the burnishing of the guide blocks. The finish of the machined hole wall is not only related to the cutting of the edge sizing blade, but also to the extrusion deformation of the guide block; the hole wall images under a microscope are shown in Figure 11.

When the guide blocks have not squeezed the hole wall, only the tool marks remain on the hole wall. When the profile edge of the outer insert is adhered to the built-up edge, it scratches against the hole wall to produce a deep groove, as shown in Figure 11a, resulting in a high hole wall roughness value. When the guide blocks are in extrusion contact with the hole wall formed by the cutting insert, the guide blocks produce an ironing effect on the hole wall, and the insert marks left on the hole wall flow plastically under the extrusion action of the guide blocks. The material migration at the peak of the hole wall fills the valley; the peak is flattened, the valley is filled, and the hole wall roughness is reduced.

**Figure 12:**
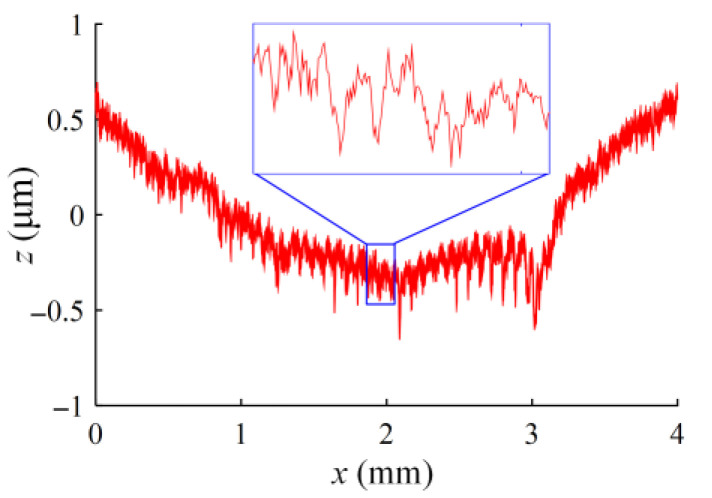
Micro-ripple curve of hole wall.

However, if the extrusion deformation of the hole wall is small, the peaks and valleys will not be completely flattened and filled, and the hole wall will still retain the tool marks that are left. The height difference between the peaks and valleys decreases, as shown in Figure 11b. If the extrusion deformation of the hole wall is suitable, the peaks and valleys will be flattened, and the roughness will be the smallest, as shown in Figure 11c. It can also be seen from the residual traces of the material flow that the guide blocks press against the hole wall circumferentially. In addition, if the extrusion deformation of the hole wall is too large, the guide blocks will leave their own traces when burnishing, forming secondary corrugations, as shown in Figure 11d, which increases the hole wall roughness. The surface microstructure curve of the hole wall is shown in Figure 12.

**Figure 13:**
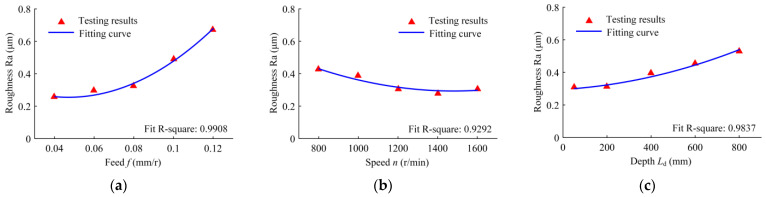
Variation in the hole wall roughness with the following drilling parameters: (**a**) the drilling feed range from 0.04 to 0.12 mm/r, when the speed is 1200 r/min and the depth is 50 mm; (**b**) the drilling speed range from 800 to 1600 r/min, when the feed is 0.08 mm/r and the depth is 50 mm; and (**c**) the drilled depth range from 50 to 800 mm, when the speed is 1200 r/min and the feed is 0.08 mm/r.

Figure 13 shows the roughness of the machined hole wall under different drilling conditions. With the increase in the feed, the hole wall roughness increases. Compared with the feed, the speed has little effect on the roughness, and the hole wall roughness decreases with the increase in the speed. As the drilling depth increases, the hole wall roughness increases.

When the feed is increased, the distance of the BTA drill along the axis of the hole wall increases for each revolution, resulting in an increase in the distance between the peaks and valleys; the extrusion density between the guide blocks and hole wall decreases, and the roughness increases. With the increase in speed, the heat generated in the drilling area increases, which leads to an increase in the temperature of the extruded contact area; the fluidity of the hole wall material is enhanced, the peak material fills the valley more easily, and the roughness is reduced. While the drilling depth increases, the extrusion contact area decreases, the extrusion contact stress increases, and the residual traces increase. As the drilling depth increases, the overhang length of the drilling tube increases, the stability of the tool system decreases, and the hole wall roughness increases.

### 3.7. Microhardness

**Figure 14:**
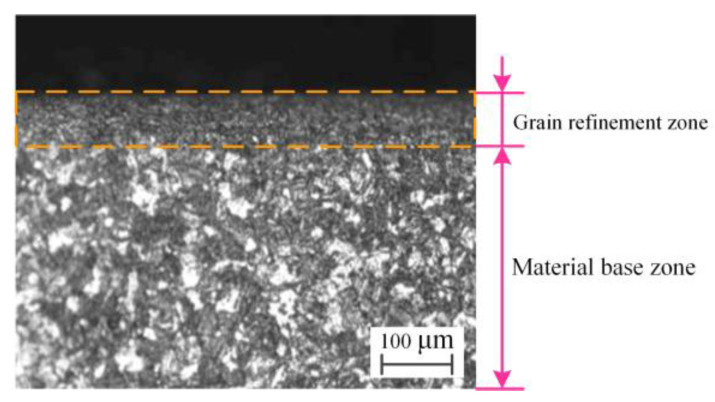
Microstructure of the drilled hole wall.

The cutting inserts cause changes in the metallographic structure of the hole wall. Then, during the secondary extrusion of the guide blocks, due to the large contact stress and friction, more heat is generated in the contact area, making the metallographic structure of the hole wall change again. The metallographic structure of the subsurface layer of the hole wall is shown in Figure 14. The subsurface layer of the hole wall has a grain refinement area, and its thickness is larger. The metal crystal structure in this region undergoes grain refinement under the conditions of plastic extrusion deformation and a high temperature to form a dense metamorphic layer with a high level of hardness [34]. The maximum temperature in the deep-hole drilling area of the BTA drill reaches 800 to 1000 °C [19], which exceeds the critical temperature of the phase transition of the metal. At the same time, the drill is in high-pressure cutting oil, and the deformation zone is quenched after the tool leaves, resulting in the higher hardness value of the hole wall.

**Figure 15:**
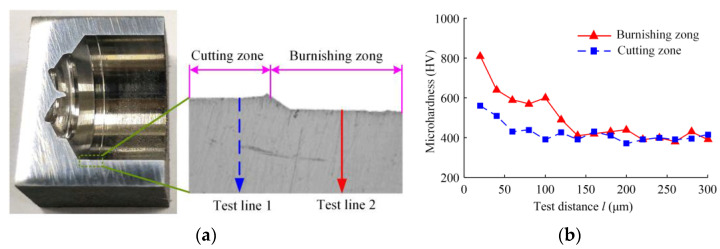
Distribution of microhardness along the tested distance: (**a**) trace line of the microhardness test point of the hole wall; and (**b**) microhardness along the tested depth.

After cutting the specimen with a drilling speed of 1200 r/min, a feed of 0.08 mm/r, and a drilling depth of 50 mm, the Vickers microhardness tester is used to detect the microhardness of the subsurface layer of the hole wall in the cutting area of the inserts and the extrusion area of the guide block at the hole’s bottom. The cutting area of the inserts is sequentially indented and measured along test line 1, while the extrusion area of the guide blocks is sequentially indented and measured along test line 2. The test results are shown in Figure 15. The maximum microhardness of the inserts’ cutting area is 1.37 times the hardness of the matrix material, and the maximum microhardness of the extrusion area is 2.15 times the hardness of the matrix material. The depth of the hardened layer after the extrusion is about 130 μm.

**Figure 16:**
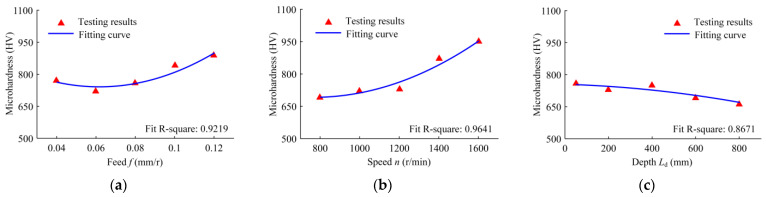
Variation in the hole wall microhardness with the following drilling parameters: (**a**) the drilling feed range from 0.04 to 0.12 mm/r, when the speed is 1200 r/min and the depth is 50 mm; (**b**) the drilling speed range from 800 to 1600 r/min, when the feed is 0.08 mm/r and the depth is 50 mm; and (**c**) the drilled depth range from 50 to 800 mm, when the speed is 1200 r/min and the feed is 0.08 mm/r.

Figure 16 shows the hole wall microhardness of the nuclear power tube plate under the conditions of different drilling feeds, rotational speeds, and drilling depths. With the increase in the feed, the microhardness of the hole wall increases; with the increase in the speed, the hole wall microhardness increases; and with the increase in the drilling depth, the microhardness of the hole wall decreases. When the feed increases, the cutting force of the inserts and the positive pressure on the guide blocks increase, the extrusion deformation of the hole wall increases, and the hole wall microhardness increases. With the increases in speed, the heat generated in the drilling deformation zone increases, which increases the depth of the grain refinement layer and the microhardness. With the increase in the drilling depth, the extruded contact area decreases, the area of the dynamic pressure oil film formed on the outer surface of the guide blocks increases, the friction between the guide blocks and the hole wall decreases, the heat generated decreases, and the microhardness decreases.

### 3.8. Residual Stress

**Figure 17:**
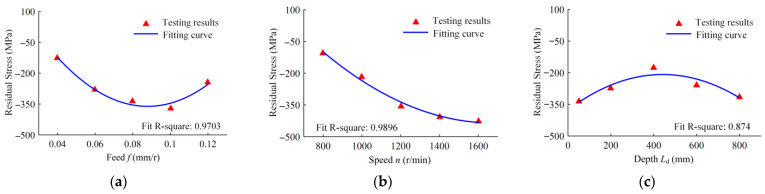
Variation in the hole wall residual stress with the following drilling parameters: (**a**) the drilling feed range from 0.04 to 0.12 mm/r, when the speed is 1200 r/min and the depth is 50 mm; (**b**) the drilling speed range from 800 to 1600 r/min, when the feed is 0.08 mm/r and the depth is 50 mm; and (**c**) the drilled depth range from 50 to 800 mm, when the speed is 1200 r/min and the feed is 0.08 mm/r.

The variation laws of the residual stress on the hole wall in relation to the feed, speed, and drilling depth are illustrated in Figure 17. The residual stress of the hole wall formed by the BTA deep-hole drilling nuclear power tube plate is compressive stress. The surface formed by the cutting inserts is tensile stress. When the guide block is burnishing, the hole wall undergoes an elastic–plastic extrusion, the workpiece material in the direction perpendicular to the hole wall is in a state of strong compression, and the hole wall is in a state of tension in the circumferential and axial directions. When the guide blocks leave the contact surface, the material rebounds, and the residual compressive stress is generated in the hole wall because the plastic deformation hinders its recovery.

When the drilling feed increases, the extrusion deformation of the hole wall increases, and the residual compressive stress increases. With the further increase in the feed, the amount of cutting per revolution increases, and the residual tensile stress generated by the cutting increases. At the same time, the point contact deformation time is reduced, and the residual compressive stress is reduced. With the increase in the speed, the drilling area temperature increases, the plastic flow of the material increases, and the residual compressive stress increases after the guide blocks leave. With the increase in drilling depth, the contact angle between the guide block and the hole wall increases, the contact area decreases, and the residual compressive stress decreases. When the drilling depth further increases, the contact stress increases, the extrusion deformation increases, and the residual compressive stress on the hole wall increases.

## 4. Conclusions

The laws of the variation in the accuracy of BTA deep-hole machining and the hole wall surface quality with the machining parameters were determined. The following conclusions were obtained.

1. The outer insert and the first guide block were severely worn; the strength of the outer insert and the contact hardness of the first guide block should be increased. The wear forms of the BTA drill mainly included diffusion wear, abrasive wear, and adhesive wear.
2. The hole diameter was greatly affected by the feed and drilling depth. With an increase in the feed and drilling depth, the diameter increased. The BTA deep-hole machining should strictly control the feed and drilling depth based on the aperture tolerance.
3. The extrusion state between the guide blocks and the hole wall directly affected the roughness. With an increase in the feed and drilling depth, the roughness increased; with an increase in speed, the roughness decreased.
4. The maximum microhardness of the hole wall was about 2.15 times that of the matrix material, and the depth of the hardened layer was about 130 μm, indicating severe machining strengthening on the hole wall. The residual stress was compressive stress. These factors can improve the fatigue and corrosion resistance of the hole wall.

## Figures and Tables

**Table 1:** Mass percentage of chemical components of SA-5083 and Inconel 690.

Material	Mn	Si	Cr	Al	S	P	V	Mo	Ti	Cu	Ni	C	Fe
SA-5083	1.4	0.32	0.21	0.02	0.015	0.015	0.03	0.052	-	-	0.75	0.16	other
Inconel 690	0.23	0.07	30.39	0.22	0.002	0.006	-	-	0.26	0.02	other	0.023	8.88

**Table 2:** Geometric parameters of ∅17.75 mm BTA drill.

Cutting Inserts	Insert Width	Approach Angle	Inclination Angle	Rake Angle	Guide Blocks	Position Angle
Central insert	5 mm	−15°	5°	−5°	1st guide block	87°
Middle insert	3.5 mm	18°	0°	0°	2nd guide block	183°
Outer insert	4 mm	18°	0°	0°		

## Data Availability

Not applicable.
